# Editorial: Advances in the imaging and treatment of valvular heart disease: “rising to the challenge”

**DOI:** 10.3389/fcvm.2023.1276202

**Published:** 2023-09-07

**Authors:** Marko Banovic, Ronak Rajani

**Affiliations:** ^1^ Belgrade Medical Faculty, University of Belgrade, Belgrade, Serbia; ^2^ Cardiology Clinic, University Clinical Center of Serbia, Belgrade, Serbia; ^3^ Cardiovascular Directorate, Guy’s and St Thomas’ NHS Foundation Trust, London, United Kingdom; ^4^ School of Biomedical Engineering and Imaging Sciences, King’s College London, London, United Kingdom

**Keywords:** valvular disese, imaging, treatment, prognosis, stratifcation

**Editorial on the Research Topic** Advances in the imaging and treatment of valvular heart disease: “rising to the challenge”

## Introduction

Valvular heart disease (VHD) is an increasing cause of cardiovascular morbidity and mortality worldwide. This overall burden is set to increase further in future years as the world's population ages and access to diagnostic techniques increase (1). Fortunately, this challenge is being met by renewed interest by the scientific community across the globe into the pathophysiology of valve disease, new potential pharmacotherapeutic agents and novel surgical and minimally invasive techniques to treat this burdensome disease. In this edition of Frontiers, the Editors have collated a series of manuscripts that detail some of the current key advancements in the diagnosis, management, and treatment across the spectrum of aortic, mitral valve and tricuspid and pulmonary valve disease (Supplementary Table S1).

## Aortic valve disease

With the rapid global expansion of transcatheter aortic valve replacement (TAVR), it was not surprising that many submissions were received detailing new deployment techniques and strategies to improve clinical outcomes. Lind et al. reported their experience with a new endovascular approach for transcatheter aortic valve replacement (TAVR) combining an axillary prosthetic conduit-based access technique with new-generation balloon-expandable TAVR prostheses. This novel approach offers hope to those patients who otherwise may have been ineligible for surgical aortic valve replacement (SAVR) or TAVR via conventional access routes. In another manuscript, Talmon-Barkar et al. investigated the interaction between transcatheter heart valve selection and valve implantation depth in 2,352 patients with a borderline basal annulus ring size. The authors showed that in these patients, the selection of larger valves resulted in reduced rates of paravalvular leak (PVL) and optimized valve hemodynamics with no increase in procedural complications. Furthermore, to refine the optimal TAVR implantation technique, Maier et al. demonstrated that a cusp-overlap deployment technique is associated with an optimized implantation depth, leading to fewer permanent conduction disturbances, but at a cost of an increase in radiation doses.

In patients with bicuspid aortic valve stenosis (AS), TAVR has been shown to be a viable treatment option. Although this is associated with increased complication rates, there is a dearth of data comparing how TAVR performs against SAVR in this patient population. To address this unknown, Gaseka et al. conducted a propensity matched cohort study evaluating patients who had undergone TAVR for bicuspid AS compared with TAVR for degenerative AS over a ten-year time frame. The authors showed that TAVR for bicuspid AS had comparable in-hospital mortality, device success, procedural complications, PVL and overall mortality compared to the degenerative AS matched cohort. There was however a higher rate of neurological complications in the TAVR bicuspid AS group. Overall, these results are encouraging but will need to be validated in one or more randomized controlled trials. For those younger patients with calcific severe AS, who are unwilling or unable to undergo SAVR, there are a lack of medical alternatives. In a thought-provoking study by Bernava et al. the potential use of shockwave ultrasound to de-calcify heart valve leaflets in a porcine model was explored. They showed that this treatment has the potential to achieve a partial debridement of calcified leaflets to improve leaflet and hydrodynamic performance. Although this preliminary work offers some hope that alternative treatments to delay the need for AVR may exist, there remains several unanswered questions relating to the use of this technology. Not least its clinical applicability and safety in humans.

There were also several papers addressing the use of imaging in the risk-stratification of patients with aortic valve disease. Kameshima et al. studied the impact of prosthesis-patient mismatch (PPM) on hemodynamics after TAVR using exercise stress-echocardiography. Perhaps unsurprisingly, the authors found that patients with PPM had a disproportionate increase in the transvalvular gradients upon stress echocardiography. This resulted in exercise-induced pulmonary hypertension and indicated a cohort with a higher NYHA functional class. This data stresses the importance of appropriate device selection and sizing in patients undergoing TAVR. Galian-Gay and associates investigated the risk of mortality and need for AVR in patients with paradoxical low-flow low-gradient (LFLG) AS. Of 1,391 patients, 147 (10.5%) had paradoxical LFLG. There was a lower need for AVR compared to a high-gradient group with a similar threshold to the normal-flow low-gradient group with no resultant differences in mortality. The authors concluded that this challenging group of patients with paradoxical LFLG AS have an intermediate clinical risk, which is in-between those patients with high-gradient AS and normal-flow LG AS.

There is increasing attention on the pathophysiology of calcified AS with recent reports suggesting that elevated serum Lipoprotein (a) is associated with the development and progression of aortic stenosis. On this theme, Liu et al. conducted a systematic review to explore this further. Eight studies with 52,931 participants were included of which four were cohort studies and four were case-control studies. The pooled results demonstrated that plasma lp(a) levels ≥50 mg/dl were associated with a 1.76-fold increased risk of calcific aortic valve disease (RR, 1.76; 95% CI, 1.47–2.11). In an experimental research Yang et al. identified ten genes and key signaling pathways through RNA-sequencing dataset and real-time PCR assay as underlying molecular targets for mechanism that may be important in AS. Both of studies indicate a hope for future targeted medical therapies for AS in the future.

## Mitral valve disease

Transcatheter mitral valve (TMV) therapies have made tremendous progress in the last decade (2, 3). Yet, in the group of patients who are eligible for TMV therapies, referral pathways remain deficient and screening failure rates high. To address this, Gill et al. report their experience in setting up a dedicated TMV clinic. The authors show how the integration of relevant experts and the “branding” of a transcatheter mitral valve service within organizations may serve to improve awareness of newer treatments and increase access to care. While TMV replacement itself remains largely in its infancy, percutaneous mitral valve edge-to-edge repair (PMVR) for treating patients with high risk/inoperable severe primary and secondary mitral regurgitation is becoming embedded into many structural interventional services. Recognizing the invaluable role of the cardiac imaging specialist to this procedure Fan et al. detail the latest advancements in transesophageal echocardiography and three-dimensional imaging, together with guide on how to apply them during PMVR procedures. Adding to our knowledge base in this area Neuser et al. demonstrated the ability of right ventricular function to improve following PMVR independent to that being seen with the left ventricle. However, this observation was attenuated in patients being referred late for treatment and in those patients with multiple comorbidities and higher surgical risk scores. This finding suggests that earlier intervention may confer improved clinical outcomes, but this needs to be confirmed in further studies. Another area of emerging interest is the use of transcatheter heart valves to treat degenerative surgical bioprosthesis. In a reasonable sized series of 26 patients Lu et al. demonstrated safety and feasibility of this procedure using the J-Valve System. In this study there was no device-related mortality, device embolization, left ventricular outflow tract obstruction, or mitral valve reintervention. The postprocedural mitral regurgitation was none or trace in all the patients and all patients were in the New York Heart Association (NYHA) class ≤ II at the last follow-up. Although this data is compelling, whether these findings can be attributed to the specific device or careful patients and imaging selection is unknown since there was no comparator device.

Rheumatic heart disease is in decline in developed countries but still affects a significant number of younger and middle-aged patients in developing countries (1). Yu and Wang. retrospectively compared 10-year survival between bioprosthetic and mechanical prosthetic valves in 1,691 middle-aged patients treated at a single center with rheumatic mitral valve disease. The authors observed no difference in all-cause mortality, as well. Certainly, as bioprosthetic valve utilization rate is increasing in recent years (4) this study demonstrates that mechanical valves are not yet to be dismissed as an option. Even more so as improvements in access to home monitoring kits increase and dedicated anticoagulation clinics expand (5).

## Tricuspid and pulmonary valve

The value of global longitudinal strain (GLS) and some of its surrogates in assessing LV function in patients with valvular diseases is well documented (6, 7). On the other hand, isolated surgical intervention to the tricuspid valve (TV) is relatively uncommon, and if performed, it should be ideally done before right ventricular (RV) dysfunction ensues. To address this issue, Kim et al. investigated the prognostic implications of biventricular global longitudinal strain in 111 patients receiving isolated tricuspid valve surgery and who underwent echocardiography before and after TV surgery. With a primary outcome being comprised of a composite of cardiovascular death, heart failure hospitalization, redo TV surgery, and heart transplantation, the authors showed an RV-GLS < 17.2% to be associated with a poor outcome during a mean follow-up of 3.8 years, and biventricular GLS < 34.0%, to be also associated with a poor prognosis. Transcatheter pulmonary valve replacement (TPVR) is a new and less invasive alternative to surgical valve replacement with acceptable long-term outcome (8). Yet, a large size transcatheter pulmonary valve could cause potential coronary artery compression and/or incomplete expansion of the stent which may increase the transvalvular gradient, and impact upon durability accelerated valve failure. To address this issue, Shang et al. explored the safety and efficacy of the Med-Zenith PT-Valve for the treatment of patients with severe pulmonary regurgitation (PR) and significantly enlarged RV outflow tract (RVOT). Successful valve implantations were achieved in all patients without noticeable device malposition, coronary artery compression, pulmonary branch obstruction or paravalvular leak. At 1-year follow-up, the RV end diastolic volume index reduced from the baseline 181.6 ± 29.0 to 123.4 ± 31.2 ml/m^2^, and the 6-min walk distance increased from 416.6 ± 97.9 to 467.8 ± 61.2 m (*p* < 0.05 for all), demonstrating both, feasibility, and efficacy for the Med-Zenith PT-Valve in the treatment of severe PR with significantly enlarged RVOT.

## Approach to valvular heart disease

Undoubtedly, there have been notable improvements in cardiac imaging techniques, multidisciplinary team structures and new devices that are making a sizeable impact on how we identify, evaluate, and treat patients with VHD. A comprehensive review by Patel et al. showcases some of the current indications and potential future roles of cardiac CT in the assessment of aortic and mitral valves for transcatheter interventions, prosthetic valve complications such as thrombosis and endocarditis, and assessment of the myocardium. Equally important is the role of the patient in shared decision making on the management of patients with VHD which is explored by Saeed et al. Here the authors focus on the importance of patient reported outcome measures based on their own experience from specialist valve clinics and emphasize how this approach may improve post-intervention quality of life, as well as maintain the efficacy of the provided treatment.

## Conclusions

The prevalence of heart valve disease across the globe is increasing and with-it cardiovascular morbidity and mortality. As our understanding of the pathophysiology of HVD increases, so does our access to advanced imaging, new surgical and transcatheter techniques and integrated patient pathways. Despite significant developments over the last twenty years, there remain several unmet needs. These relate to better methods of identifying asymptomatic patients, predicting symptom onset and the development and implementation of new pharmacotherapeutic agents and medical devices. As we approach an era where computer processing power and artificial intelligence is reaching the clinical domain, the next twenty years are likely to witness a quantum leap in how we approach and manage patients with heart valve disease. If 10 years ago we thought that the future came quickly (9), then it will seem mild compared to how quickly new advances are coming to us.
