# Supplementary material for: Editorial: Advances in the imaging and treatment of valvular heart disease: “rising to the challenge”
Source: Front Cardiovasc Med. 2023 Sep 7;10:1276202. doi: 10.3389/fcvm.2023.1276202 (PMC10513464; doi:10.3389/fcvm.2023.1276202)
Supplement: Supplementary file 1 [file Table1.doc]

Table: Summary of accepted papers with main findings and future challenges

| Paper by: | Main findings | Challenges to be addressed in the future |
| --- | --- | --- |
| Lind et al. | Novel approach for TAVR combining an axillary prosthetic conduit-based access technique with new-generation balloon-expandable TAVR prostheses | Due to the limited sample-size feasibility and efficacy needs to be confirmed in larger series and with other valve types. The mid and long-term follow-up are needed. |
| Talmon-Barkar et al. | In borderline cases, the selection of larger valves resulted in reduced rates of paravalvular leak and optimized valve hemodynamics with no increase in procedural complications | Potential impact of certain confounding factors on study outcomes (such as sinus valsalva diameter or calcium score) should be re-evaluated |
| Maier et al. | a cusp-overlap deployment technique is associated with an optimized implantation depth, leading to fewer permanent conduction disturbances | Further procedure refinement in order to limit radiation doses during procedure |
| Gaseka et al. | TAVR for bicuspid AS had comparable in-hospital mortality, device success, procedural complications, PVL and overall mortality compared to the degenerative AS matched cohort | Higher rate of neurological events after TAVR in bicuspid AS group. Furthermore, results have to be validated in one or more randomized controlled trials |
| Bernava et al. | Use of shockwave ultrasound to de-calcify heart valve leaflets. Partiel debridement reached. | Several unanswered questions relating to the use of this technology. Study needs feasibility and safety confirmation in humans |
| Kameshima et al. | PPM after TAVR resulted in exercise-induced pulmonary hypertension and indicated a cohort with a higher NYHA functional class | Only balloon-expandable THV were used, the Self-expandable THVs and comparison with prosthetic valve types is warranted. |
| Galian-Gay et al. | In patients with paradoxical low-flow low-gradient AS here is a lower need for AVR compared to a high-gradient group with a similar threshold to the normal-flow low-gradient group and with no differences in mortality | Prospective, randomized trial is warrented |
| Liu et al. | Plasma lp(a) levels ≥50 mg/dL were associated with a 1.76-fold increased risk of calcific aortic valve disease | The therapeutic consequences, and does lowering Lp(a) and to what extent brings a clinical benefit, needs to be investigated |
| Cheng et al. | Authors found ten genes and key signaling pathways through RNA-sequencing dataset and realtime PCR assay as underlying molecular targets in AS | Results indicate a hope for future targeted medical therapies for AS |
| Gill et al. | Integration of relevant experts and the “branding” of transcatheter mitral valve service within organizations may serve to improve awareness of newer treatments and increase access to care | Further evaluation of these models is needed to confirm this hypothesis |
| Fan et al. | Details latest advancements in transesophageal echo and threedimensional imaging, together with guide on how to apply them during percutaneous mitral valve procedures | Further rechnological improvements in 3D imaging on resolution and frame rates are awaiting, as well as the advent of full-volume 3D intracardiac echocardiography technique |
| Neuser et al. | Improvement in right ventricular function following PMVR is independent to that being seen with the left ventricle | Study results suggests that earlier intervention may confer improved clinical outcomes, but this needs to be confirmed in further studies |
| Lu et al. | Authors demonstrated safety and feasibility of the J-Valve System to treat degenerative surgical bioprosthesisthis procedure | Although this data is compelling, whether these findings can be attributed to the specific device or careful patients and imaging selection is unknown since there was no comparator device |
| Yu et al. | There were no difference in 10-year survival between bioprosthetic and mechanical prosthetic valves in middle-aged patients treated to the rheumatic mitral valve disease | Prospective studied are warraned, as well as in degenerative mitral and aortic valve diseases |
| Kim et al | RV-GLS < 17.2% is associated with a poor outcome during a mean follow-up of 3.8 years in patients receiving isolated tricuspid valve surgery | A larger and more comprehensive prospective multicenter studies of patients with isolated TR are needed |
| Shang et al. | Demonstrated safety and efficacy of the Med-Zenith PT-Valve for the treatment of patients with severe pulmonary regurgitation and significantly enlarged RV outflow tract | Studies with longer follow-up, as well as with patient swith advanced heart failure due to the RV volume overload are needed |
| Patel et al. | Authors discuss indications and potential future roles of cardiac CT in the assessment of aortic and mitral valves for transcatheter interventions and prosthetic valve complications | Fusion imaging using a CT and photon counting CT might herald a new era in cardiac CT, enabling safer transcatheter valvular procedures, and improve signal to noise ratio, reducing artifacts and radiation. Its integration into clinical use may improve the utility of CT for valvular heart disease |
| Saeed et al. | Authors emphasize the importance of patient reported outcome measures and how this approach may improve post-intervention quality of life, as well as maintain the efficacy of the provided treatment | Further research focusing on values and preferences of patients with VHD, particularly AS undergoing SAVR vs TAVI, as well as overall valve intervention vs. conservative treatment, is warranted |
| Aleksandric et al. | Authors discuss myocardial revascularization strategy in patients with severe aortic stenosis undergoing TAVR | To determine the optimal FFR and iFR ischemic thresholds in patients with severe AS and co-existing CAD, additional prospective randomized trials are needed with a larger number of patients. Furthermore, the prognostic relevance of PCI before or after TAVR remains controversial |
